# Comparison of the characteristics of long-term users of electronic cigarettes versus nicotine replacement therapy: A cross-sectional survey of English ex-smokers and current smokers

**DOI:** 10.1016/j.drugalcdep.2015.05.005

**Published:** 2015-08-01

**Authors:** Victoria A. Nelson, Maciej L. Goniewicz, Emma Beard, Jamie Brown, Kate Sheals, Robert West, Lion Shahab

**Affiliations:** a Department of Epidemiology and Public Health, University College London, London, UK; b Department of Health Behavior, Roswell Park Cancer Institute, Buffalo, NY, USA; c Department of Clinical, Educational and Health Psychology, University College London, London, UK

**Keywords:** NRT use, Electronic cigarettes, Harm reduction, Identity, Smoking cessation, Nicotine withdrawal

## Abstract

**Background:**

Electronic cigarettes (ECs) and nicotine replacement therapy (NRT) are non-combustible nicotine delivery devices being widely used as a partial or a complete long-term substitute for smoking. Little is known about the characteristics of long-term users, their smoking behaviour, attachment to smoking, experience of nicotine withdrawal symptoms, or their views on these devices. This study aimed to provide preliminary evidence on this and compare users of the different products.

**Methods:**

UK participants were recruited from four naturally occurring groups of long-term (≥6 months) users of either EC or NRT who had stopped or continued to smoke (*N* = 36 per group, total *N* = 144). Participants completed a questionnaire assessing socio-demographic and smoking characteristics, nicotine withdrawal symptoms, smoker identity and attitudes towards the products they were using.

**Results:**

Adjusting for relevant confounders, EC use was associated with a stronger smoker identity (Wald *X*^2^(1) = 3.9, *p* = 0.048) and greater product endorsement (Wald *X*^2^(1) = 4.6, *p* = 0.024) than NRT use, irrespective of smoking status. Among ex-smokers, EC users reported less severe mood and physical symptoms (Wald *X*^2^(1) = 6.1, *p* = 0.014) and cravings (Wald *X*^2^(1) = 8.5, *p* = 0.003), higher perceived helpfulness of the product (Wald *X*^2^(1) = 4.8, *p* = 0.028) and lower intentions to stop using the product (Wald *X*^2^(1) = 17.6, *p* < 0.001) than NRT users.

**Conclusions:**

Compared with people who use NRT for at least 6 months, those who use EC over that time period appear to have a stronger smoker identity and like their products more. Among long-term users who have stopped smoking, ECs are perceived as more helpful than NRT, appear more effective in controlling withdrawal symptoms and continued use may be more likely.

## Highlights

• E-cigarette (EC) and nicotine replacement therapy (NRT) are used for harm reduction.
• But little comparative data on their long-term use in smokers and quitters exist.
• We find that long-term EC users have stronger smoker identities than NRT users.
• EC users rate their product more highly and have lower intentions to stop use.
• In long-term quitters, withdrawal symptoms are reduced in EC compared with NRT user.

## 1 Introduction

Despite the huge burden of tobacco smoking on health (Doll et al., 2004; WHO, 2012), smokers struggle to quit successfully and global smoking prevalence remains stubbornly high (Eriksen et al., 2012). Stopping smoking is largely difficult because of the highly addictive properties of nicotine (Watkins et al., 2000). Nicotine withdrawal produces both physical symptoms (e.g., tremors) and mood symptoms (e.g., elevated anxiety), and causes the majority of smokers making an unassisted quit attempt to return to smoking within two weeks (Hughes et al., 2004). Thus, nicotine withdrawal may be a useful target to support long-term transitions to smoking reduction or complete smoking cessation. This is the rationale for the provision of medicinal nicotine in the form of nicotine replacement therapy (NRT), which has been shown in randomised trials to increase quit rates by 50 to 70% (Stead et al., 2012). However, beyond smoking cessation, for smokers who are unwilling or unable to quit, NRT use for harm reduction may be a valuable strategy in reducing the burden of tobacco use, and in the UK, guidelines recommend this approach for these smokers (NICE, 2013). As the combustion of cigarettes is recognised as the primary cause of cigarette toxicity, harm reduction in this context is defined as the use of non-combustible forms of nicotine delivery to partially or fully replace combustible forms such as cigarettes in the long run (Le Houezec et al., 2011). Research suggests that a substantial minority of smokers use NRT for long-term harm reduction, e.g. for temporary abstinence or to cut-down on cigarettes, and that this may be increasing (Beard et al., 2011; Hammond et al., 2008; Levy et al., 2007; Silla et al., 2014).

In addition to traditional NRT, electronic cigarette (EC) is another non-combustible nicotine delivery device which has gained a wide popularity in recent years (Brown et al., 2014b; King et al., 2013; Vardavas et al., 2014) and potentially may be particularly suited for harm reduction, given high levels of dual use in the population (McMillen et al., 2014) and continued long-term single and dual use in clinical trials (Shahab and Goniewicz, 2014). EC usually consists of a battery, heating element, and a tank or a cartridge containing a nicotine solution (‘e-liquid’). The battery is typically activated either manually or by inhalation through the device, and produces an aerosol that can be inhaled by the user. Although some toxic chemicals have been detected in EC aerosol (Goniewicz et al., 2013; Schober et al., 2014; Vardavas et al., 2012), it does not contain tar, or most of the other chemical compounds detected in cigarette smoke, as the process does not involve combustion, resulting in levels of toxicants at least an order of magnitude lower than that in cigarette smoke (Goniewicz et al., 2014; Kosmider et al., 2014). Thus EC can arguably be considered a much safer alternative to smoking cigarettes (Hajek et al., 2014). ECs have also been demonstrated to increase cessation rates in clinical trials (McRobbie et al., 2014) and some population studies (Biener and Hargraves, 2015; Brown et al., 2014a), but not all observational studies have detected an effect (Grana et al., 2014), and more research is needed to confirm EC effectiveness, using an appropriate methodology to distinguish between the impact of EC use on cessation when used as part of a quit attempt vs. when it is used for any general purpose (Hajek et al., 2014).

Although some concerns remain in the population regarding the safety of prolonged use of non-combustible nicotine delivery devices (Black et al., 2012; Dockrell et al., 2013), the evidence indicates that long-term NRT use is safe in terms of levels of nicotine delivered (Shahab et al., 2014) and associated toxicity (Benowitz and Gourlay, 1997; Hubbard et al., 2005) and growing data on EC would suggest the same (Hajek et al., 2014). This, combined with the known toxicity of combustible nicotine use, further supports the idea of harm reduction, shifting smokers towards non-combustible nicotine delivery devices and away from smoked tobacco. However, relatively little is known about the processes which underpin a smoker's transition to sole use of non-combustible nicotine delivery devices and whether long-term use of such products aids cessation or maintains smoking in the long run.

One universal mechanism worth investigating in the context of understanding this transition is “smoker identity”, the self-concept that being a smoker is an essential constituent of one's identity (Shadel and Mermelstein, 1996; Shadel et al., 1996). It has been posited that identity influences behaviour by creating strong wants or needs, such as wanting to be a non-smoker, which compete with external impulses, such as the desire to smoke, and may therefore reinforce or undermine shifts in behaviour (West and Brown, 2013). Studies have observed a weakening in smoker identity during cessation as smokers distanced themselves from an unwanted smoker identity (Johnson et al., 2003; Vangeli and West, 2012), and liking being a smoker has been identified as an important barrier to smoking cessation (Tombor et al., 2013; van den Putte et al., 2009).

Another important factor in the transition from smoker to non-smoker is the physiological impact of cessation. The role of mood and physical symptoms in relapse is well-documented (West et al., 1989), and even after long periods of abstinence the presence of withdrawal symptoms has been shown to predict return to smoking (Piasecki et al., 2003). In line with existing theory (West and Brown, 2013), it is therefore important that such symptoms are minimised to ensure the motivation not to smoke remains stronger than the motivation to smoke. For this reason, effective harm reduction should treat negative mood and physical symptoms.

Lastly, attitudes towards the product, e.g., in terms of satisfaction or intention to stop its use, are likely to inform its suitability for long-term harm reduction purposes, on the one hand, and transition towards complete cessation of all nicotine products, on the other. Ideally, all factors that are likely to influence the product–contingent transition from smoking to non-smoking would be assessed prospectively. However, given the length of time needed to evaluate the use of non-combustible nicotine products for harm reduction appropriately, this study used a pragmatic approach, purposively selecting participants who had been using products for at least six months.

In order to evaluate the transitions from smoking to non-smoking, both smokers and ex-smokers using non-combustible nicotine delivery devices were selected. In addition, comparisons were made between EC and NRT users to determine the relative associations with the modality of nicotine delivery. Given the relative lack of data on EC, NRT was deemed a useful comparator as it has well-established effectiveness. Specifically, the present study assesses the associations between smoking status and product type among long-term users of EC or NRT with (1) smoker identity, (2) withdrawal symptoms, and (3) attitudes towards non-combustible nicotine delivery devices.

## 2 Methods

### 2.1 Study design and procedure

This cross-sectional study forms part of a larger, international study assessing the impact of long-term use of non-combustible nicotine delivery devices on health (currently being prepared for publication). The present study, which focuses on psychological measures collected only in the UK sub-sample, also involved the collection of biological samples (not reported here) as well as administration of a questionnaire at a single laboratory appointment, lasting approximately 30 min. Smokers and ex-smokers using either EC or NRT on a long-term basis of at least six months were purposively recruited, resulting in four groups of participants: current and ex-smokers using NRT and current and ex-smokers using EC. Participants were screened into these four naturally occurring groups to allow for comparisons between EC and NRT use, and between smoking status. Participants were reimbursed for time and travel. The study received ethical approval from the University College London (UCL) Ethics Committee (Project ID 0483/002).

### 2.2 Participants

Participants were told that this study was about the effects of long-term use of non-combustible nicotine delivery devices and recruited in the greater London, UK area during January–July, 2014 using various recruiting methods to access a diverse sample. These included adverts in newspapers, Facebook, online electronic cigarette forums, posters in independent pharmacies, emails to students and staff at UCL, the use of an online smokers panel as well as marketing companies.

Participants were screened for eligibility via phone or online questionnaires. Inclusion criteria were based on long-term product use in order to control for a noted learning curve in effective EC use (e.g., Bullen et al., 2013). Ex-smokers had to have quit any tobacco products (including waterpipe, cigars, smokeless products) for six months, use their non-combustible nicotine delivery device weekly for the past six months, and not use other non-combustible nicotine delivery devices regularly (i.e., ex-smoker NRT users could not use EC regularly and vice versa). Smokers had to smoke an average of one cigarette per day and meet the same non-combustible nicotine delivery device use criteria as ex-smokers. Current smoking status was verified using a breathalyser to assess expired air carbon-monoxide (CO); readings above 10 ppm indicated current smoking. Due to the collection of biological samples (not reported here), participants were excluded if they were younger than 18 years old, had a history of heart or lung disease, were pregnant, or had bleeding gums, illness, or infection within 24 h of their scheduled appointment.

Thirty-six participants were recruited into each of the four study groups which provided sufficient power to detect a medium-sized effect on outcome measures (Cohen's *d* = 0.40, see Kraemer and Kupfer, 2006). Data for all participants (*N* = 144) are provided in Table 1.

### 2.3 Measures

#### 2.3.1 Outcome measures

Based on work underlining the validity of simple measures of smoker identity (e.g., Tombor et al., 2013), the present study used an established item to determine smoker identity strength (Shadel and Mermelstein, 1996): participants were asked to rank their agreement with the statement, ‘Smoking is a part of me’ on a Likert scale of 1 (‘not at all’) to 5 (‘completely agree’).

Withdrawal symptoms were assessed with the validated Mood and Physical Symptoms Scale (MPSS, West and Hajek, 2004; West et al., 2006) which assesses cravings (two items; cigarette craving strength and frequency; range 0–5 per item, from ‘no urges’/‘not at all’ to ‘extremely strong’/‘all the time’) and other general mood and physical symptoms related to withdrawal (seven items; being irritable, restless, depressed, hungry, anxious, subjected to poor sleep, poor concentration; range 1–5 per item, from ‘not at all’ to ‘extremely’).

Attitudes towards NRT or EC were assessed with three measures. Intention to stop product use was measured using a modified version of the motivation to stop scale (Kotz et al., 2013), replacing the term ‘cigarette’ with ‘e-cigarette’ or ‘NRT’, and with higher values indicating greater motivation to stop use (seven response options; ranging from ‘I don’t want to stop’ to ‘I really want to stop and intend to in the next months’). Using 5-point Likert scales, participants were further asked whether they found the product helpful in enabling them to refrain from smoking, with response options ranging from ‘not at all helpful’ to ‘extremely helpful’ and whether they would recommend the product to a friend who wanted to stop smoking, with response options ranging from ‘definitely not’ to ‘definitely’.

#### 2.3.2 Covariates

Standard socio-demographic and smoking characteristics, including age, sex, ethnicity, education, length of current/past smoking, current or past cigarettes smoked per day, cigarette dependence, motivation to stop, number of quit attempts were also measured. In addition, a number of product use characteristics were assessed such as length and frequency of product use (see Table 1). Participants were asked to indicate the length of use, latency to use the product in the morning as an indicator of dependence and consumption. The latter was assessed by asking NRT users to indicate the strength and the type of the product used and quantity used per day, week, or month. EC users were also asked about the type of the product they used; those using first generation EC (disposable/re-chargeable) and those using second or third generation EC (refillable or advanced personal vaporisers) were asked to indicate, respectively, either the nicotine content of the disposable/cartridge or the concentration of the e-liquid used as well as the quantity used per day, week, or month. Please refer to the supplementary information for the full questionnaire.

### 2.4 Analysis

**Fig. 1:**
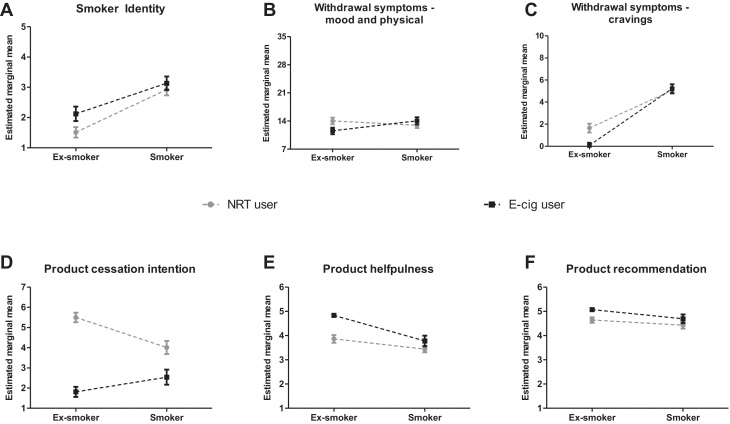
Association of smoking status and product type with smoker identity (A), withdrawal symptoms (B, C) and attitudes towards the products (D–F); Estimated marginal means are adjusted for age, gender, ethnicity, marital status, education, length of smoking, number of cigarettes smoked per day, length of product use, product consumption level and latency to product use; error bars are SEM; grey circles denote nicotine replacement therapy (NRT) users; black squares indicate electronic cigarette (EC) users.

Analyses were conducted with SPSS Version 21.0. Simple associations between study groups and continuous demographic variables, smoking characteristics, and product use characteristics were assessed with one-way ANOVAs or independent *t*-tests, and categorical variables were assessed with chi-square analysis, controlling for family-wise error rate using the false discovery rate (Benjamini and Hochberg, 1995) and for multiple comparisons using the Sidak correction in post hoc analysis. Generalised linear models were used to assess main or interaction effects of product use (EC versus NRT) and smoker status (ex- versus current smoker) on smoker identity, withdrawal symptoms, and attitudes towards the product, controlling for relevant covariates (as listed in Fig. 1).

## 3 Results

Compared with the UK general population (Office for National Statistics, 2012), the present sample was younger, and more likely to be white, male, educated, and cohabiting or married (Table 1). Cigarette consumption reflected national data (e.g., Fidler et al., 2011) and participants had smoked for nearly 20 years on average and had used either NRT or e-cigarettes for about one--and-a-half years. Ex-smokers had also stopped for about one-and-a-half years and had significantly lower levels of CO than current smokers (Table 1). The four groups were balanced among the majority of socio-demographic and smoking characteristics measured. However, there were significantly more male smokers using EC than smokers using NRT, and more cohabiting smokers using NRT than EC (Table 1). As would be expected, current cigarette consumption was lower among dual users than past cigarette consumption reported by ex-smokers and four out of five smokers reported trying to cut down cigarette consumption. Amongst smokers, NRT users reported having made more recent quit attempts and being more motivated to quit than EC users (Table 1). While there were no differences in terms of the length of product use, EC users reported greater daily nicotine consumption than NRT users, and ex-smokers generally had a shorter latency to product use in the morning than smokers (Table 1).

### 3.1 Associations with smoker identity

In order to control for possible confounding influences, all socio-demographic, smoking, and product use characteristics with data available for all groups were included as covariates in further analysis. Generally, a stronger smoker identity was associated with greater current or past cigarette consumption (Wald *X*^2^(1) = 4.6, *p* = 0.031) and with being female (Wald *X*^2^(1) = 7.6, *p* = 0.006). However, there was no interaction of smoking status by the product type (Wald *X*^2^(1) = 1.1, ns). As would be expected, there was a main effect of smoking status on smoker identity (Wald *X*^2^(1) = 29.5, *p* < 0.001): current smokers expressed a stronger smoker identity than ex-smokers. There was also a main effect of the product type (Wald *X*^2^(1) = 3.9, *p* = 0.048): smoker identity was more pronounced among EC users than NRT users, irrespective of smoking status and other covariates (see Fig. 1A).

### 3.2 Associations with withdrawal symptoms

In terms of withdrawal symptoms, higher current/past cigarette consumption (Wald *X*^2^(1) = 8.7, *p* = 0.003) and being female (Wald *X*^2^(1) = 4.5, *p* = 0.034) were associated with more pronounced mood and physical withdrawal symptoms in this sample. In addition, there was a significant interaction of product type and smoking status (Wald *X*^2^(1) = 6.1, *p* = 0.014). As shown in Fig. 1B, while there was no product-dependent difference among smokers, ex-smokers who use NRT reported higher mood and physical withdrawal symptoms than ex-smokers using EC. These findings are largely mirrored when looking at reported cravings. As before, there was a significant product type by smoking status interaction, such that NRT use was associated with greater cravings only among ex- but not current smokers (Wald *X*^2^(1) = 8.5, *p* = 0.003, Fig. 1C). In addition, the results indicated that lower product use (as measured by average daily nicotine intake derived from products) was associated with stronger cravings (Wald *X*^2^(1) = 6.8, *p* = 0.009).

### 3.3 Associations with attitudes towards products

Non-white participants in this sample were more likely to consider stopping the use of non-combustible nicotine delivery devices (Wald *X*^2^(1) = 6.2, *p* = 0.013) as were those participants who had used products for longer (Wald *X*^2^(1) = 9.4, *p* = 0.002). In addition, there was also a clear product type by smoking status interaction on intention (Wald *X*^2^(1) = 17.6, *p* < 0.001): whilst NRT users were generally more likely to intend to stop using their product than EC users, this difference was significantly stronger among ex-smokers than smokers (see Fig. 1D). Similarly, product type interacted with smoking status on the perceived helpfulness of the product (Wald *X*^2^(1) = 4.8, *p* = 0.028). ECs were generally rated as more helpful for keeping off cigarettes than NRT but again, this difference was significantly stronger among ex-smokers than smokers (Fig. 1E). Lastly, there were main effects of product type (Wald *X*^2^(1) = 4.6, *p* = 0.032) and smoking status (Wald *X*^2^(1) = 5.1, *p* = 0.024) for recommending the product to others but no interaction (Wald *X*^2^(1) = 0.4, ns). EC users or ex-smokers were significantly more likely to recommend the product as an aid to smoking cessation than NRT users or current smokers (Fig. 1F).

## 4 Discussion

The long-term use of e-cigarettes compared with licensed NRT by ex- and current smokers is associated with a stronger smoker identity and product endorsement. Among ex-smokers only, EC as compared with NRT use is associated with lower withdrawal symptoms, greater perceived helpfulness of the product for stopping smoking and weaker intention to stop product use.

As in this study, previous work suggests that smoker identity may play a role in product use and smoking status (Tombor et al., 2013; Vangeli and West, 2012). Given e-cigarette users had a stronger smoker identity than NRT users irrespective of whether they smoked or had stopped smoking, the present results support the common-sense assumption that ECs have a particular appeal for those who identify more strongly with smoking. This may be due to a greater similarity between smoking cigarettes and vaping and could also reflect the possibility that EC may be viewed as a consumer product for recreational use whereas NRT is seen as a medicinal product for treatment purposes. Alternatively, it could be that EC and NRT users do not differ initially but that EC use sustains smoker identity or that NRT use undermines this identity over time. This cross-sectional study cannot distinguish these possibilities.

Nicotine craving and mood and physical withdrawal symptoms were virtually non-existent among ex-smokers using EC and significantly lower than among ex-smokers using NRT. While previous research indicates both NRT (Moore et al., 2009; Stead et al., 2012) and EC (Hajek et al., 2014; McRobbie et al., 2014) can be useful for cessation and harm reduction purposes, our study suggests that in experienced users EC may be especially effective at reducing nicotine withdrawal. Given that this sample comprises long-term users, this effect is unlikely to be the result of incorrect product use. Notwithstanding the adjustment for smoking characteristics in the analysis, this result may also again reflect self-selection (i.e., ex-smokers who use EC may have had lower dependence to start off with and thus would experience less withdrawal).

ECs were rated as more helpful for stopping smoking than NRT by ex-smokers using these products. EC users, in particular, ex-smokers, were consequently less likely than NRT users to intend to stop using the product. In addition, motivation to stop smoking and the number of past year quit attempts were greater among smokers who concurrently used NRT than EC. Taken together, these findings are consistent with a gradual transition towards a non-smoker identity among long-term NRT users who smoke (Silla et al., 2014) and reflect a possible identity shift among ex-smokers which may involve the want to be free of any nicotine products (Vangeli and West, 2012).

On the one hand, findings are encouraging insofar as they suggest that EC could be a powerful harm reduction tool, at least as effective as the established NRT, and that they may be particularly helpful in engaging those smokers who are not motivated to quit and/or strongly identified as smokers. Indeed, it has been reported that a minority of long-term ex-smokers maintain a strong smoker identity (Vangeli et al., 2010). For these ex-smokers, in particular, EC may enable complete substitution of combustible with non-combustible nicotine delivery devices. On the other hand, it is possible that if a stronger smoking identity is maintained by EC use, this may undermine long-term outcomes as establishing a firm non-smoker identity may be important to resist relapse to smoking (Tombor et al., 2015). However, such speculations need to be tested in experimental design.

This study has several limitations which restrict the conclusions that can be drawn from the present study. First, although diverse recruitment methods were used, the sample was purposively selected and thus findings may not generalise to the general population. However, relevant confounders were controlled for to reduce selection bias, and the distribution of participant characteristics was roughly similar to those found in larger, broadly representative studies of non-combustible nicotine delivery devices (Brown et al., 2014b; Silla et al., 2014). Second, due to the cross-sectional design, it is not possible to determine the direction of the association between product choice and outcome variables as these may be due to self-selection. While a prospective design would be preferable, given the relative novelty of EC and the associated lack of data on this topic, we chose this pragmatic design to pin-point important associations with long-term use now which can be investigated further in longitudinal studies. Third, although smoking status was verified and validated self-report measures were used, these may not be able to capture fully complex concepts such as smoker identity.

In light of these findings, future research should continue to explore and clarify the association of smoker identity, withdrawal symptoms and attitudes towards the products with long-term use of NRT and EC among smokers and ex-smokers. In particular, it would be important to establish whether smoker identity and intentions to stop nicotine use influence product choice or whether product use impacts the strength of smoker identity and the decision to stop nicotine use completely. Notwithstanding the potential of self-selection bias given the cross-sectional nature of data, the observed interactions of product use with smoking status are consistent with EC being a particularly suitable harm reduction tool to switch smokers from combustible tobacco to permanent non-combustible nicotine use whereas NRT may be more suitable as harm reduction tool in the short-to-intermediate term.

In conclusion, long-term EC use is associated with a stronger smoker identity and positive attitudes towards the product than the long-term NRT use. ECs are generally perceived as more helpful than NRT for stopping smoking by ex-smokers and may be more effective at reducing withdrawal symptoms. Based on self-reported intention to stop product use, NRT compared with EC may also be more likely to result in complete cessation of nicotine among long-term users who have stopped smoking.

## Role of funding source

We are grateful to Cancer Research UK for funding the study (C27061/A16929). E.B., J.B., A.Mc., L.S. and R.W. are members of the UK Centre for Tobacco and Alcohol Studies. K.S. is funded by a CRUK Lynn MacFadyen Scholarship (C27061/A18679). J.B.’s post is funded by the Society for Study of Addiction. E.B. is funded by CRUK and the National Institute for Health Research (NIHR)’s School for Public Health Research (SPHR). The views are those of the authors(s) and not necessarily those of the funders. The funders had no involvement in the design of the study, collection, analysis or interpretation of the data, the writing of the report, or the decision to submit the paper for publication.

## Contributors

L.S. conceived this study and contributed to the write-up. L.S. takes full responsibility for the integrity of the data and the accuracy of the data analysis. V.N. had full access to all the data in the study and wrote the initial draft. K.S. and V.N. collected the data and M.G., E.B., J.B. and R.W., contributed to the write-up of the manuscript.

## Conflict of interest

L.S. has received a research grant and honoraria for a talk and travel expenses from a Pfizer, manufacturer of smoking cessation medications. M.L.G. received a research grant from Pfizer, manufacturer of smoking cessation medications. J.B. and E.B. have both received an unrestricted research grant from Pfizer to study population trends in smoking. R.W. has received travel funds and hospitality from, and undertaken research and consultancy for, pharmaceutical companies that manufacture or research products aimed at helping smokers to stop. V.N. and K.S. have no competing interests.

## Figures and Tables

**Table 1:** Socio-demographic, smoking and product use characteristics by study group.

	Total (*N* = 144)	EC users		NRT users	
		Ex-smoker (*N* = 36)	Smoker (*N* = 36)	Ex-smoker (*N* = 36)	Smoker (*N* = 36)
**Socio-demographics**					
Mean age (SD)	38.6 (11.0)	38.5 (11.1)	39.3 (13.1)	40.3 (11.1)	36.4 (8.5)
% Male (N)	61.8 (89)	80.6 (29)a	69.4 (25)a	58.3 (21)a,b	38.9 (14)b
% White (N)	70.1 (101)	83.3 (30)	75.0 (27)	63.9 (23)	58.3 (21)
% Married/cohabiting (N)	42.4 (61)	33.3 (12)a,b	27.9 (10)a	61.1 (22)b	47.2 (17)a,b
% University degree (N)	56.2 (81)	50.0 (18)	50.0 (18)	58.3 (21)	66.7 (24)

**Smoking characteristics**					
Mean length of smoking, years (SD)	18.9 (11.1)	19.0 (11.5)	21.5 (12.2)	17.1 (10.5)	18.0 (10.1)
Mean cigarettes per day^a^ (SD)	13.5 (8.6)	16.5 (8.0)a	11.9 (9.6)a,b	14.7 (10.3)a,b	10.8 (4.6)b
Mean length stopped smoking, months (SD)	18.1 (17.5)	19.7 (15.7)	–	16.5 (19.2)	-
Mean FTCD (SD)	3.0 (2.1)	–	3.1 (1.9)	–	2.8 (2.2)
% Currently trying to cut down	84.5 (60)	–	80.0 (28)	–	88.9 (32)
Mean MTTS (SD)	4.5 (1.8)	–	3.9 (2.1)a	–	5.1 (1.4)b
Mean number of quit attempts in the past year (SD)	1.3 (1.7)	–	0.8 (1.0)a	–	1.7 (2.0)b
Mean CO level in ppm (SD)	5.5 (5.4)	2.8 (1.1)a	8.3 (5.5)b	3.3 (3.2)a	7.7 (7.2)b

**Product**^b^**use characteristics**					
Mean length of product use, months (SD)	16.6 (17.0)	20.1 (15.4)	14.5 (9.1)	15.8 (24.8)	16.0 (15.0)
Mean product use, nicotine mg/day (SD)	21.0 (25.9)	32.6 (34.1)a	20.5 (33.0)a,b	17.0 (15.6)a,b	13.1 (7.8)b
% Use product within 1 h of waking (N)	56.9 (82)	80.6 (29)a	44.4 (16)b	63.9 (23)a,b	38.9 (14)b

FTCD – Fagerstrom test of cigarette dependence (Fagerstrom 2012); MTSS – Motivation to stop scale (Kotz et al., 2013); NRT – nicotine replacement therapy; EC – electronic cigarette; CO – carbon-monoxide. Different letters between groups indicate significant differences at *p* < 0.05.

a Current consumption for smokers, past consumption for ex-smokers.

b Product’ refers to NRT or EC.
