# Supplementary material for: Comparison of the characteristics of long-term users of electronic cigarettes versus nicotine replacement therapy: A cross-sectional survey of English ex-smokers and current smokers
Source: Drug Alcohol Depend. 2015 Aug 1;153:300–5. doi: 10.1016/j.drugalcdep.2015.05.005 (PMC4528068; doi:10.1016/j.drugalcdep.2015.05.005)
Supplement: Supplementary file 1 [file mmc1.pdf]

Background information	
1. Age	___________ years
2. Sex	Male 1<br>Female 2
3. Ethnic group	**White**<br>British[1] Irish[2] Other White[3]<br>**Black**<br>African[4] Caribbean[5] Other Black[6]<br>**Asian**<br>Indian[7] Pakistani[8] Bangladeshi[9] Chinese[10]<br>Other Asian[11]<br>**Mixed**<br>White/Black[12] White/Asian[13] Other Mixed[14]<br>**Other**[15]<br>(Please specify) :__________________________
4. What is your highest qualification?	None 1<br>GCSE or equivalent 2<br>A level or equivalent 3<br>Degree or equivalent 4<br>Other 5
5. Marital Status	Single 1<br>Separated/divorced 2<br>Married/living with partner 3<br>Other 4
6. What percentage of your friends and family smoke?	_________________ (enter number)
7. In the last 3 weeks have you had signs of any of the following *(Circle as many as apply)*	Chest infection 1<br>Cold/Flu 2<br>Sore Throat 3<br>Fever 4<br>Nothing wrong 0

8. Have you experienced any of the following during the last 30 days and if so, please indicate how strong this effect was.

	**Unpleasant effect**	**Strength *(circle one number)***			
		Nothing at all	*Weak*	*Moderate*	*Strong*
	Dizziness	1	2	3	4
	Headache	1	2	3	4
	Nausea	1	2	3	4
	Sleep disturbances	1	2	3	4
	Concentration difficulties	1	2	3	4
	Seizures	1	2	3	4
	Visual disturbances	1	2	3	4
	Trouble with breathing during the day	1	2	3	4
	Trouble with breathing when sleeping	1	2	3	4
	Wheezing	1	2	3	4
	Chest pain	1	2	3	4
	Chest tightness	1	2	3	4
	Cough during the day	1	2	3	4
	Cough during the night	1	2	3	4
	Dry mouth	1	2	3	4
	Extensive saliva	1	2	3	4
	Other, please specify:<br>_________________________________	1	2	3	4

9. Over the last 24h have you taken any of these medications *(Circle as many as apply)*	Aspirin 1<br>Paracetamol 2<br>Ibuprofen 3<br>Flu vaccine 4<br>Nothing 0<br>Other, please specify:<br><br>______________________	
**Smoking**		
10. "Smoking is part of me" *(please circle number to indicate how much you agree with the statement)*	**Not at all** 1 2 3 4 5 **Completely agree**	
11. What brand of cigarettes do you usually smoke?		
12. How many cigarettes per day do you usually smoke? *(Please write exact number on the line and then circle the appropriate number)*	Number of cigarettes/day:_______________<br><br>10 or less 0<br>11 to 20 1<br>21 to 30 2<br>31 or more 3	
13. Do you smoke…. ?	Daily 1<br>Less than daily 2	
14. "I could be a non-smoker" *(please circle number to indicate how much you agree with the statement)*	**Not at all** 1 2 3 4 5 **Completely agree**	
15. How long ago did you smoke your most recent cigarette?	Date:<br>Time:	
16. Do you smoke hand-rolled cigarettes?	Never 0<br>Sometimes 1<br>Exclusively 2	
17. How many do you usually smoke per day?		
18. How much tobacco do you usually use per week?	______________ g *or* ______________ ounces	
19. At what age did you start smoking on a daily basis?	______________ years	
20. Are you currently trying to cut down on how much you smoke but not currently trying to stop?	Yes 1<br>No 2	
21. Which of these products, if any, have you used in to help you cut-down?	Zyban 1<br>24 h patch 2<br>16 h patch 3<br>4mg gum 4<br>2mg gum 5<br>Inhalator 6<br>Nasal spray 7	Oral spray 8<br>Microtab 9<br>Lozenge 10<br>Champix 11<br>E-cigarette 12<br>None 13
22. Do you regularly use any of the following in situations when you are not allowed to smoke?	Zyban 1<br>24 h patch 2<br>16 h patch 3<br>4mg gum 4<br>2mg gum 5<br>Inhalator 6<br>Nasal spray 7	Oral spray 8<br>Microtab 9<br>Lozenge 10<br>Champix 11<br>E-cigarette 12<br>None 13

23. How many serious attempts to stop smoking have you made in the last 12 months? By serious attempt I mean you decided that you would try to make sure you never smoked again *(Please write number in box*)	
24. Which, if any, of the following contributed to you making your most recent quit attempt (*tick all that apply*)?	Advice from a GP\health professional 1<br>TV advert for a nicotine replacement product 2<br>TV advert for an electronic cigarette 3<br>Government TV\radio\press advert 4<br>Hearing about a new stop smoking treatment 5<br>A decision that smoking was too expensive 6<br>Being faced with smoking restrictions 7<br>I knew someone else who was stopping 8<br>Seeing a health warning on a cigarette packet 9<br>Being contacted by my local NHS SSS 10<br>Health problems I had at the time 11<br>A concern about future health problems 12<br>Attending a local stop smoking activity or event 13<br>Something said by family\friends\children 14<br>A significant birthday 15<br>Other, please specify:<br>________________________
25. During your most recent serious quit attempt which (if any) did you try to help you stop smoking (*tick all that apply*)?	Zyban 1<br>24 h patch 2<br>16 h patch 3<br>4mg gum 4<br>2mg gum 5<br>Inhalator 6<br>Nasal spray 7<br>Oral spray 8<br>Microtab 9<br>Lozenge 10<br>Champix 11<br>E-cigarette 12<br>NHS SSS 13<br>Helpline 14<br>Book/Booklet 15<br>Website 16<br>App on handheld device 17<br>Hypnotherapy 18<br>Acupuncture 19<br>None 20<br>Other, please specify:<br>________________________
26. If applicable, how long ago was the last time you used products to help you stop smoking? (*Please write number in box and indicate years, months, weeks or days)*	
27. How long have you been smoking for? *(in years)*	
28. In the past year, what is the longest you have gone without a cigarette? *(Please write number in box and indicate years, months, weeks or days)*	
29. How soon after you wake up do you smoke your first cigarette?	Within 5 minutes 3<br>6-30 minutes 2<br>31-60 minutes 1<br>More than 60 minutes 0
30. Do you find it difficult to stop smoking in no-smoking areas?	No 0<br>Yes 1
31. Which cigarette would you hate most to give up?	The first of the morning 1<br>Other 0

32. Do you smoke more frequently in the first hours after waking than during the rest of the day?	No 0<br>Yes 1
33. Do you smoke if you are so ill that you are in bed most of the day?	No 0<br>Yes 1

34. Which of the following best describes you?	I REALLY want to stop smoking and intend to in the next month	1
	I REALLY want to stop smoking and intend to in the next 3 months	2
	I want to stop smoking and hope to soon	3
	I REALLY want to stop smoking but I don't know when I will	4
	I want to stop smoking but haven't thought about when	5
	I think I should stop smoking but don't really want to	6
	I don't want to stop smoking	7
35. Compared with e-cigarettes, do you think that cigarettes are…?	Completely safe	1
	Less dangerous than e-cigarettes	2
	As dangerous as e-cigarettes	3
	More dangerous than e-cigarettes	4
	Don't know	0
36. Compared with NRT, do you think that cigarettes are…?	Completely safe	1
	Less dangerous than NRT	2
	As dangerous as NRT	3
	More dangerous than NRT	4
	Don't know	0
37. Compared with e-cigarettes, do you think that cigarettes are…?	Not at all addictive	1
	Less addictive than e-cigarettes	2
	As addictive as e-cigarettes	3
	More addictive than e-cigarettes	4
	Don't know	0
38. Compared with NRT, do you think that cigarettes are…?	Not at all addictive	1
	Less addictive than NRT	2
	As addictive as NRT	3
	More addictive than NRT	4
	Don't know	0
39. Do you think you are addicted to cigarettes?	Yes	1
	No	0

E-cigarettes (if applicable)

40. What is the brand name and model of e-cigarette you are currently using?	_______________________________	
41. What is the solvent (base) of the nicotine solution (e-liquid) in the cigarette you usually use?	Only propylene glycol (PG)	1
	Only glycerine (e.g. VG)	2
	Mixture of glycerine and propylene glycol (VG/PG)	3
	I don't know	4
42. What kind of e-cigarette do you currently use most often?	Disposable model	1
	With replaceable cartridge	2
	Tank system – refill with nicotine solution	3
	Other (Please describe ______________)	4
43. What is the nicotine concentration in the liquid or cartridges you use?	___________ mg/ml	

44. What size are the cartridges you use?	___________ ml
45. How many cartridges do you usually use?	___________ per day *or* ___________ per week
46. How long ago did you last use an e-cigarette?	Date:<br>Time:
47. In the past year, what is the longest you have gone without using an e-cigarette? *(Please write number)*	___________ days/weeks/months *(delete as appropriate)*
48. How long have you been using e-cigarettes? *(in months)*	
49. Where did you buy your first e-cigarette?	Online 1<br>Kiosk in a shopping mall 2<br>Special shop (vapour lounge) 3<br>Other 4
50. What was the brand and model of e-cigarette you used first time?	______________________________
51. What kind of e-cigarette did you use first time?	Disposable model 1<br>With replaceable cartridge 2<br>Tank system – refill with nicotine solution 3<br>Other (Please describe ______________) 4
52. Where did you buy the e-cigarette you are currently using?	Online 1<br>Kiosk in a shopping mall 2<br>Special shop (vapour lounge) 3<br>Other 4
53. Where do you currently buy cartridges for e-cigarette or e-liquid?	Online 1<br>Kiosk in a shopping mall 2<br>Special shop (vapour lounge) 3<br>Other 4
54. Was the last e-cigarette you tried or used flavoured to taste like menthol, mint or any other flavour such as spice, candy fruit or alcohol?	Yes 1<br>No 0
55. What is the flavour of e-cigarette you usually use?	__________________________________
56. Usually, how soon after waking up do you draw your first puff on your e-cigarette?	0 to 5 minutes 1<br>6 to 15 minutes 2<br>16 to 30 minutes 3<br>31 to 60 minutes 4<br>61+ minutes 5
57. For you, stopping your use of the e-cigarette for good would be…	Impossible 1<br>Very difficult 2<br>Fairly difficult 3<br>Fairly easy 4<br>Very easy 5
58. Which of the following best describes you?	I REALLY want to stop using e-cigs and intend to in the next month 1<br>I REALLY want to stop using e-cigs and intend to in the next 3 months 2<br>I want to stop using e-cigs and hope to soon 3<br>I REALLY want to stop using e-cigs but I don't know when I will 4<br>I want to stop using e-cigs but haven't thought about when 5<br>I think I should stop using e-cigs but don't really want to 6<br>I don't want to stop using e-cigs 7

59. Compared to tobacco cigarettes how satisfying is the e-cigarette?	Much less than usual<br>A little less than usual<br>The same as usual<br>A little more than usual<br>Much more than usual	1<br>2<br>3<br>4<br>5
60. How helpful do you find e-cigarettes in enabling you to keep from smoking?	Not at all helpful<br>Slightly helpful<br>Somewhat helpful<br>Very helpful<br>Extremely helpful	1<br>2<br>3<br>4<br>5
61. How pleasant is the e-cigarette to use?	Not at all pleasant<br>Slightly pleasant<br>Somewhat pleasant<br>Very pleasant<br>Extremely pleasant	1<br>2<br>3<br>4<br>5
62. Do you enjoy the sensation in your throat and chest after using the e-cigarette?	Not at all<br>Slightly<br>Somewhat<br>Very<br>Extremely	1<br>2<br>3<br>4<br>5
63. How embarrassing are e-cigarettes to use in the company of others?	Not at all embarrassing<br>Slightly embarrassing<br>Somewhat embarrassing<br>Very embarrassing<br>Extremely embarrassing	1<br>2<br>3<br>4<br>5
64. Would you use e-cigarettes to help you to stop smoking?	Not a smoker<br>Definitely not<br>Probably not<br>Maybe<br>Probably<br>Definitely	0<br>1<br>2<br>3<br>4<br>5
65. Would you recommend e-cigarettes to a friend who wanted to stop smoking?	Definitely not<br>Probably not<br>Maybe<br>Probably<br>Definitely	1<br>2<br>3<br>4<br>5
66. Do you think e-cigarettes are safe for your health?	Yes, absolutely safe<br>No, but they are safer than tobacco cigarettes<br>No, they are as dangerous as tobacco cigarettes<br>No, they are more dangerous than tobacco cigarettes	1<br>2<br>3<br>4
67. Do you think e-cigarettes are addictive?	No, absolutely not<br>Yes, but they are less addictive than tobacco cigarettes<br>Yes, they are as addictive as tobacco cigarettes<br>Yes, they are more addictive than tobacco cigarettes	1<br>2<br>3<br>4
68. Do you think you are addicted to e-cigarettes?	Yes<br>No	1<br>0
69. Which product do you like the most?	E-cigarette<br>Tobacco cigarette	1<br>2

70. Which product is easier to use?	E-cigarette 1<br>Tobacco cigarette 2
71. Which product is more embarrassing to use?	E-cigarette 1<br>Tobacco cigarette 2

Nicotine Replacement Therapy (if applicable)

72. What kind of NRT did you first start using?	Patches 1<br>Gum 2<br>Inhalator 3<br>Nasal spray 4<br>Mouth spray 5<br>Microtab 6<br>Lozenge/tablet 7		
73. What type of NRT are you currently using?			
74. How much of this NRT do you usually use? *(State the amount of nicotine in one 'item' of the relevant NRT and the frequency of usage in days, weeks or months; <u>check material brought in</u>)*		Mg per item	No per day/wk/mth
	Patches<br>Gum<br>Inhalator<br>Nasal spray<br>Mouth spray<br>Microtab<br>Lozenge/tablet		
75. How long ago did you last use NRT?	Date:<br>Time:		
76. In the past year, what is the longest you have gone without using NRT? *(Please write number in box and indicate years, months, weeks or days)*			
77. How long have you been using NRT? *(in months)*			
78. Usually, how soon after waking up do you first use NRT?	0 to 5 minutes 1<br>6 to 15 minutes 2<br>16 to 30 minutes 3<br>31 to 60 minutes 4<br>61+ minutes 5		
79. For you, stopping your use of NRT for good would be…	Impossible 1<br>Very difficult 2<br>Fairly difficult 3<br>Fairly easy 4<br>Very easy 5		

80. Which of the following best describes you?	I REALLY want to stop using NRT and intend to in the next month	1
	I REALLY want to stop using NRT and intend to in the next 3 months	2
	I want to stop using NRT and hope to soon	3
	I REALLY want to stop using NRT but I don't know when I will	4
	I want to stop using NRT but haven't thought about when	5
	I think I should stop using NRT but don't really want to	6
	I don't want to stop using NRT	7

81. Compared to tobacco cigarettes how satisfying is NRT?	Much less than usual<br>A little less than usual<br>The same as usual<br>A little more than usual<br>Much more than usual	1<br>2<br>3<br>4<br>5
82. How helpful do you find NRT in enabling you to keep from smoking?	Not at all helpful<br>Slightly helpful<br>Somewhat helpful<br>Very helpful<br>Extremely helpful	1<br>2<br>3<br>4<br>5
83. How pleasant is NRT to use?	Not at all pleasant<br>Slightly pleasant<br>Somewhat pleasant<br>Very pleasant<br>Extremely pleasant	1<br>2<br>3<br>4<br>5
84. Do you enjoy the sensation in your throat and chest after using NRT?	Not at all<br>Slightly<br>Somewhat<br>Very<br>Extremely	1<br>2<br>3<br>4<br>5
85. How embarrassing is NRT to use in the company of others?	Not at all embarrassing<br>Slightly embarrassing<br>Somewhat embarrassing<br>Very embarrassing<br>Extremely embarrassing	1<br>2<br>3<br>4<br>5
86. Would you use NRT to help you to stop smoking?	Not a smoker<br>Definitely not<br>Probably not<br>Maybe<br>Probably<br>Definitely	0<br>1<br>2<br>3<br>4<br>5
87. Would you recommend NRT to a friend who wanted to stop smoking?	Definitely not<br>Probably not<br>Maybe<br>Probably<br>Definitely	1<br>2<br>3<br>4<br>5
88. Do you think NRT is safe for your health?	Yes, absolutely safe<br>No, but it is safer than tobacco cigarettes<br>No, it is as dangerous as tobacco cigarettes<br>No, it is more dangerous than tobacco cigarettes	1<br>2<br>3<br>4
89. Do you think NRT is addictive?	No, absolutely not<br>Yes, but it is less addictive than tobacco cigarettes<br>Yes, it is as addictive as tobacco cigarettes<br>Yes, it is more addictive than tobacco cigarettes	1<br>2<br>3<br>4
90. Do you think you are addicted to NRT?	Yes<br>No	1<br>0

Life Satisfaction

These questions ask about your overall happiness and satisfaction with life. Please indicate how much you agree with the following questions/statements by circling the number that corresponds most to how you feel.

91. All things considered, how satisfied are you with your life as a whole? *(Circle one number)*

Very dissatisfied	Moderately dissatisfied	No feelings either way	Moderately satisfied	Very satisfied
1	2	3	4	5

92. Some people are generally very happy. They enjoy life regardless of what is going on, getting the most out of everything. To what extent does this characterisation describe you? *(Circle one number)*

Not at all	1	2	3	4	5	6	7	A great deal

Mood and Physical Symptoms

These questions ask you to describe how you have been feeling over the past 24 hours and about any urges to use particular products you might have had. Please indicate how much you agree with the following questions/statements by circling the number that corresponds most to how you feel.

93. Please show for each of the items below how you have been feeling over the past 24 hours. (*Circle one number for each item).*

	Not at all	Slightly	Somewhat	Very	Extremely
1. Depressed	1	2	3	4	5
2. Anxious	1	2	3	4	5
3. Irritable	1	2	3	4	5
4. Restless	1	2	3	4	5
5. Hungry	1	2	3	4	5
6. Poor concentration	1	2	3	4	5
7. Poor sleep at night	1	2	3	4	5

94. How much of the time have you felt the urge to smoke a ***cigarette*** in the past 24 hours? *(Circle one number)*

Not at all	A little of the time	Some of the time	A lot of the time	Almost all the time	All the time
0	1	2	3	4	5

95. How strong have the urges been? *(Circle one number)*

No urges	Slight	Moderate	Strong	Very strong	Extremely strong
0	1	2	3	4	5

96. How much of the time have you felt the urge to use ***nicotine replacement therapy/e-cigs*** in the past 24 hours? *(Circle one number)*

Not at all	A little of the time	Some of the time	A lot of the time	Almost all the time	All the time
0	1	2	3	4	5

97. How strong have the urges been? *(Circle one number)*

No urges	Slight	Moderate	Strong	Very strong	Extremely strong
0	1	2	3	4	5
